# Self-Sensing Polymer Composite: White-Light-Illuminated Reinforcing Fibreglass Bundle for Deformation Monitoring

**DOI:** 10.3390/s19071745

**Published:** 2019-04-11

**Authors:** Gergely Hegedus, Tamas Sarkadi, Tibor Czigany

**Affiliations:** 1 Department of Polymer Engineering, Faculty of Mechanical Engineering, Budapest University of Technology and Economics, Muegyetem rkp. 3, H-1111 Budapest, Hungary; hegedus@pt.bme.hu; 2 Department of Atomic Physics, Faculty of Natural Sciences, Budapest University of Technology and Economics, Budafoki ut. 8, H-1111 Budapest, Hungary; sarkadi@eik.bme.hu; 3 MTA–BME Research Group for Composite Science and Technology, Muegyetem rkp. 3, H-1111 Budapest, Hungary

**Keywords:** reinforcing glass fibre, light transmission, deformation monitoring, multifunctional composite, self-sensing polymer composite

## Abstract

The goal of our research was to develop a continuous glass fibre-reinforced epoxy matrix self-sensing composite. A fibre bundle arbitrarily chosen from the reinforcing glass fabric in the composite was prepared to guide white light. The power of the light transmitted by the fibres changes as a result of tensile loading. In our research, we show that a selected fibre bundle even without any special preparation can be used as a sensor to detect deformation even before the composite structure is damaged (before fibre breaking).

## 1. Introduction

The production of composites is increasing every year, and the technological development of fibre-reinforced polymer composites is especially fast. Thanks to the great engineering freedom of combining materials, composites are not only used for small unique products but also for large mass-produced products with great load-bearing capacity in different industries (e.g., the automotive industry or energetics) [1].

Composite products often operate in extreme conditions. Their usability is mostly determined by accidental damage, aging and corrosion. The most dangerous defect of composite structures is fracture, which is always preceded by cracking and crack propagation. Designing composite parts and simulating their behaviour still poses challenges [2,3], and there is not enough information for experimental structural solutions. Therefore, diagnostic tests for composite structural materials play an increasingly important role [4,5,6]. Removing and reinserting the part to be tested takes time and energy, which can be saved with a well-designed testing method using a built-in sensor, and the defect can be predicted and prevented. Since the sensor is built into the part, it can continuously provide a signal during operation, and continuous structural health monitoring can be performed by processing the signals. There are ready-made solutions for structural health monitoring with built-in optical sensors (e.g., Fiber Bragg Grating sensors [7,8,9,10,11,12,13]) with precise measurement of temperature and deformation. Although the diameter of the optical sensor is quite small (typically 125 microns), it is still an order of magnitude larger than the diameter of the individual reinforcing fibres, which creates an inhomogeneity in the composite, and a resin-rich area is formed next to the sensor, which can be a starting point of failure [14]. Because the refractive indexes of resins vary in a wide range [15], a glass fibre-reinforced composite can be made capable of transmitting light with specially prepared glass fibres built into a resin with a refractive index lower than that of the glass fibres [16,17,18,19,20,21], and the change in the intensity of the transmitted light can indicate damage to the composite structure. This phenomenon can be used for structural health monitoring; this makes another sensor—which impairs the integrity of the structure—unnecessary. Using the reinforcement of the composite as a sensor results in a multifunctional self-sensing composite [22,23,24].

With our research, we set out to prove that an arbitrarily chosen reinforcing fibre bundle of a commercially available glass fibre fabric without special preparation can be used not only to indicate damage in the composite structure but also to show the deformation of a composite with a general-purpose epoxy matrix, when the fibre bundle is illuminated with white light. The purpose of our article is to prove our assumption that the power of light leaving the end of the illuminated fibre bundle is greatly influenced by the connection between crossing fibres in the fabric. Compression of the illuminated fibre bundle and the fibre bundles crossing it increases the amount of light coupled out of the illuminated bundle sideways, and this phenomenon can be exploited to indicate loading (reversibly) before the individual fibres of the fibre bundle break—that is, before the failure of the composite structure. Another advantage of the procedure compared to built-in sensors is that the use of a reinforcing fibre bundle influences the mechanical properties of the composite structure the least.

## 2. Materials and Methods

### 2.1. Materials Used, Manufacturing the Specimens

The E-glass reinforcement used in the experiments was plain weave (0°/90°) (weft direction: 400 tex; warp direction: 300 tex) and had a refractive index of 1.56, a density of 2.54–2.60 g/cm^3^, and an area density of 320 g/m^2^ ± 6% (STR 014-320-125, Krosglass, Poland). We used the reinforcement without further special surface preparation. The reinforcing fibre bundle can transmit light if the medium surrounding it has a lower refractive index, therefore we needed a resin with a refractive index lower than that of glass to manufacture the specimens. Most resin datasheets do not include the refractive index of the resin, so we measured the refractive index of several resin systems [20]. Based on the results, we applied the MR3012 epoxy resin with a refractive index of 1.52 (Ipox chemicals, Germany) and the MH3122 curing agent (Ipox chemicals, Germany). The mixing ratio was 100:40 by mass. This glycerine-based three-function general-purpose epoxy resin can be produced from potentially renewable resources and can also be used with injection technologies.

**Figure 1:**
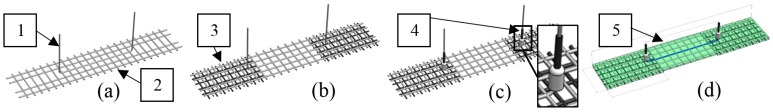
The steps of making a multifunctional composite specimen: (**a**) The selected fibre bundle (1) pulled out of the fabric (2) at both ends; (**b**) laying the second layer of reinforcement (3) on both sides; (**c**) putting the ends of the selected fibre bundle in cord-end terminals (4); and (**d**) the specimen soaked with resin (5).

An important criterion during the manufacturing of the specimens and laying the fibres was that the specimen must be suitable for the coupling of light and proper clamping (even under external load). Another aspect was that the fibre bundle should be continuously under moderate tensile stress within the specimen and that the start and end of the bundle should enclose a certain angle so that the direction of light exiting the fibres does not coincide with the direction of the axis of the fibres. As a result, the unwanted effect of direct light coming from the light source and the light transmitted by the resin can be eliminated. Our goal was to produce a multifunctional composite specimen, and we focused on a selected fibre bundle of the continuous reinforcement of the structure—an organic part of the reinforcement, not an element inserted into the reinforcement later (Figure 1).

**Figure 2:**
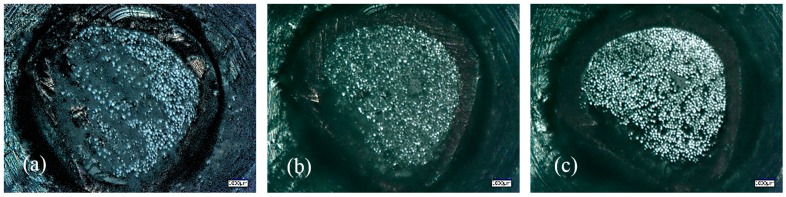
Microscope images of (**a**) a fibre bundle held together after cutting, and (**b**) 30-µm (**c**) and 0.2-µm fineness polishing.

The two ends of a selected fibre bundle were taken out of one layer of a 120-mm-wide (0,90) weave reinforcing fabric, so that 50 mm of the bundle remained in the fabric (Figure 1a), and the ends of the bundle were inserted in cord-end terminals (Figure 1c). The ends of the fibre bundle were equidistant from the edges of the fabric, as shown in Figure 1a. Two additional layers of 40-mm-wide reinforcing fabric were laid on the fabric 40 mm from each other, as shown in Figure 1b. The space between them was between the cord-end terminals, symmetrically, at equal distance from the terminals. This way, the specimen was reinforced at the place of clamping, where the bundle came out, and this ensured controlled failure in the area between the cord-end terminals. The fabric prepared in this way was soaked with epoxy resin (Figure 1d). After curing, the specimens were cut from the composite sheet as 25-mm-wide strips. The ends of the fibre bundles in the cord-end terminals were polished to the required quality. Before polishing, we cut the end of the fibre bundle that extended beyond the cord-end terminal. The resin residue stuck to the side of the cord-end terminal was removed and the ends of the fibre bundle were consecutively polished smooth with polishing paper of 30, 6, 3, 1, and 0.2 µm fineness (Figure 2).

**Figure 3:**
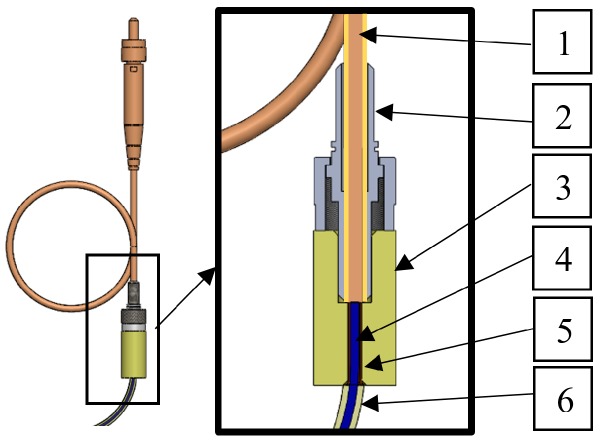
The connection of the fibre bundle in the cord-end terminal with the optical fibre in the standard subminiature assembly (SMA) connector (1: polymer optical fibre; 2: SMA connector; 3: the unique connector we developed; 4: reinforcing fibre bundle; 5: uninsulated cord-end terminal; 6: resin).

Coupling the light into the fibre bundle and transmitting the output light at the other end of the bundle can be done with an optical fibre so that both the light source and the signal-processing unit can be in a fixed position, and if necessary, further away from the specimen. In this case, the diameter of the core of the optical fibre had to be larger than the diameter of the fibre bundle, so that all the elementary fibres of the bundle were within the core of the optical fibre. This way, all fibres were illuminated by the light on the end that the light entered the bundle. It was the same on the end where the light exited the bundle; all the light from all the fibres should be picked up by the optical fibre. The core diameter of glass optical fibres is typically far smaller than the diameter of the fibre bundle, but among polymer optical fibres (POFs) there are fibres with a large enough diameter. A disadvantage of polymer optical fibres is their higher attenuation, but in the short distances in these measurements, this did not influence the test results within the wavelength range of illumination. The outer diameter of the polymer optical fibre used in the experiments (Tru Components, VD-1500, Germany) was 1500 ± 90 µm, and its core diameter was 1470 ± 90 µm. The refractive index of the core was 1.492, its smallest bending radius was 20 mm, its numerical aperture was 0.5, and its specific attenuation at 650 nm was <220 dB/km. Its operating temperature range was −50 °C to 70 °C. We put a standard subminiature assembly (SMA) connector on both ends of the optical fibre to connect it to the light source and the signal processing unit. There is no commercially available device to connect a cord-end terminal and an SMA connector, so we developed a unique connector. The connector was cylindrical and had a counterbored hole in its axis. The hole with smaller diameter held the uninsulated cord-end terminal in place with an accuracy of 0.1 mm, while the larger hole positioned the SMA connector with an accuracy of 0.01 mm; this ensured that the individual fibres were all within the cross section of the polymer optical fibre in the SMA connector (Figure 3).

### 2.2. Equipment and Measurement Methods

Our goal was to show that a mechanical load of the composite material had a significant effect on the optical transmittance of the fibre bundles. Therefore, we had to choose an efficient light source and a sensitive detector to illuminate the fibre bundles and detect the transmitted light. We used a white LED light source (Cree, XLamp, XP-C LEDs, USA) in our tests in order to prove that these commercially available light sources are suitable to implement our fibre monitoring concept. The wide spectral bandwidth of the LED [25] makes the light source universal, because various types of fibre–matrix systems with different transmission properties can be analysed by the same light source.

As a detector, we used a photodiode (Hamamatsu, S1133-01, Japan) because of its low noise and high sensitivity in the whole visible spectrum. Based on the datasheet of the detector [26], we could claim that the current characteristic of the incident light power detector was linear in the measurement range we used, thus we supposed that the current of the detector current was proportional to the incident light power. During our measurements, we made an effort to keep the equipment at a constant temperature of 24 °C in order to stabilize the spectrum of the light source and the sensitivity of the detector.

The relative decrease of transmitted power (*RTP_x_*) was evaluated during the measurements according to Equation (1):

*RTP_x_* = (*P_0_−P_x_*)/*P_0_*, (1)

where *P_0_* is the transmitted light power before mechanical loading, and *P_x_* is the transmitted light power at a given mechanical loading.

In the following, the change of these relative values can be found as percentages. Three specimens were made for each measurement. The following sections only show the results of a typical measurement. The specimens were subjected to a tensile test in a tensile tester (Zwick, BZ020/TN2S, Germany). The deformation of the specimens was calculated from the displacement of the crosshead of the tensile tester. The measurement layouts are shown in the Chapter 3.

## 3. Results

Our first goal was to experimentally prove that the compression of the crossing bundles of the reinforcing glass fabric decreased the white light transmission of the illuminated bundle. Therefore, the load on the specimens was perpendicular to the plane of the fabric. In this case, the crossing fibre bundles got closer to each other, and the guided light of the bundle was scattered out of the fibre surface with higher probability than when the fibre bundle was unloaded.

### 3.1. The Effect of Compression Perpendicular to the Plane of the Fabric

**Figure 4:**
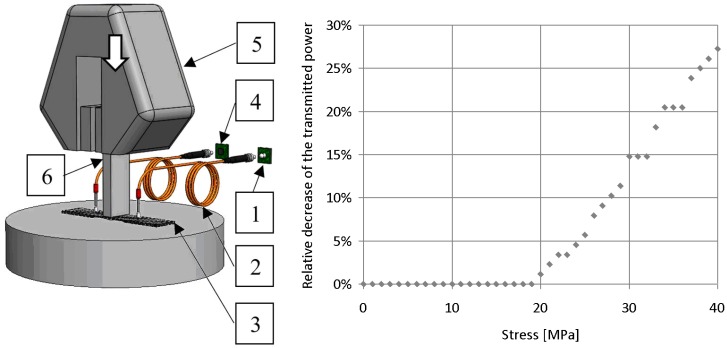
Compression measurement layout (1: light source, 2: signal transmitter (polymer optical fibre), 3: specimen, 4: photodiode, 5: clamp of the tensile tester, 6: compression fixture) and a typical plot.

We manufactured specimens as in Section 2.1. and put them in the tensile tester to load the specimens in the direction perpendicular to the plane of the fabric, as shown in Figure 4. The loaded fibre bundle was illuminated, and the power of transmitted light was measured. The maximum compression force was 10,000 N, uploading speed was 10 N/s, and the compressed area was 10 mm × 25 mm.

The relative change of transmitted light power as defined by Equation (1) is plotted as a function of the stress in Figure 4. The diagram shows that the transmittance of the fibre bundle did not change significantly at stresses below 20 MPa. This is because up to this load, the matrix around the fibre bundle deformed and took the load itself. The surfaces of the specimens were not completely smooth, thus the compression fixture initially loaded the highest point of the specimen rather than the whole area of the illuminated fibre bundle. After the first period of loading, transmitted light power decreased significantly. The relative decrease of transmitted light power was approximately proportional to the stress. In this loading period, the compression fixture produced increasing stress on the specimen area where the illuminated bundle was located. The result indicates that the compression stress perpendicular to the plane of the E-glass fabric can be detected by the proposed light guide glass fibre illuminated by white light.

### 3.2. The Effect of a Tensile Load Parallel to the Axis of the Fibre Bundle

In the previous subsection we saw that the compression stress perpendicular to the sheet of the E-glass fabric had an effect on the light transmission properties of the fibre bundles. Because of compression, the fibres got closer to each other in the fabric, and light scattering increased on the surface of the illuminated fibres. This scattering caused a loss in the power of the transmitted light, which could be efficiently detected.

It is easy to see that if the composite had a tensile load parallel to the fibre bundles, the bundles crossing each other in the fabric also got closer, modifying the light transmission properties of the fibres. Below, we analyse the effect of the tensile load parallel to the fibre bundle on the light transmission of the E-glass.

**Figure 5:**
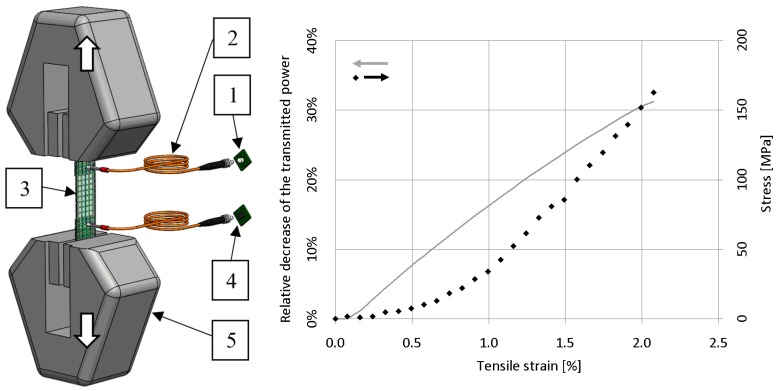
Tensile measurement layout (1: light source, 2: signal transmitter (polymer optical fibre), 3: specimen, 4: photodiode, 5: clamp of the tensile machine) and a typical plot.

In the tensile test, pulling speed was 1 mm/min while we measured the transmitted light power at the end of the illuminated fibre bundle (Figure 5).

The relative decrease of the transmitted light power and the stress are plotted on the diagram in Figure 5 as a function of tensile strain. The measurement results prove that the light power transmitted by the fibre bundle changed monotonically as a function of the tensile load. We can recognise that the change of the transmitted power–tensile strain function was not linear; it had an increasing slope. This means that the illuminated fibre bundle as a stress detector had enhanced sensitivity in the tensile range higher than 1%. This is advantageous, because it allows critical stresses approaching the break to be accurately detected.

### 3.3. The Effect of Repeated Loads

**Figure 6:**
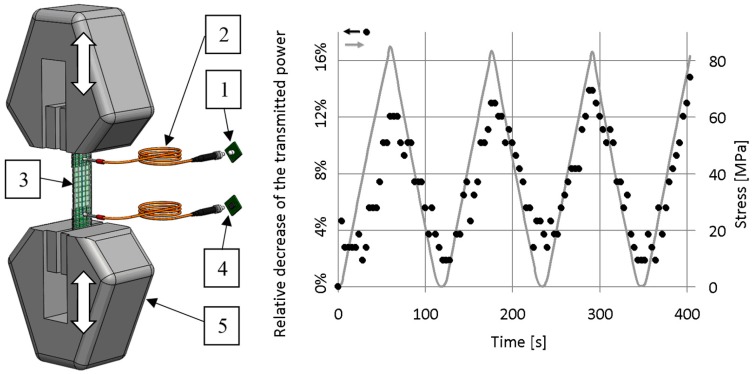
Cyclic tensile measurement layout (1: light source, 2: signal transmitter (polymer optical fibre), 3: specimen, 4: photodiode, 5: clamp of the tensile machine) and a typical plot.

Next, we analysed whether the transmittance modulation of the glass fibres was reversible or caused by irreversible effects, such as fibre breaking. Therefore, we examined the change of the transmitted light power of the selected fibre bundle effected by a periodically changing tensile load (Figure 6). The measurement layout is described in Section 3.2. The rate of increasing and decreasing the load was 1 mm/min, up to a displacement of 0.8 mm. This displacement corresponded to a strain of 1%. Clamping length was 80 mm.

The measurement results show that the relative decrease of the transmitted light power followed not only the increasing but also the decreasing periods of the tensile load. Based on these facts, supposing some approximations, we can claim that the change in the transmission of the bundle is reversible.

Since the power of the transmitted light returned to a value close to the original value after loading was ceased, it is certain that the change in transmitted light power during loading was not caused by fibre breaking or some other irreversible effect. Therefore, with the help of a fibre bundle made to transmit light, smaller mechanical stresses can be detected efficiently in the composite; these stresses do not cause the fibres to break and can be reversed.

## 4. Conclusions

In this paper, we proposed a health monitoring technique for glass-fabric-reinforced composites. This method involved preparing a fibre bundle of the reinforcement fabric to guide light. We showed that the light transmission abilities of the fibre bundle changed when mechanical load was applied on the composite material. 

We proved that a general-purpose, commercially available resin of lower refractive index could be applied for light guiding, even without special preparation, and its surface treatment layer did not need to be removed either. Based on this, a self-sensing composite could be produced with the reinforcing material. Health could be monitored without any significant modification of the composite structure.

We found that commercially available white light sources could be used to illuminate the glass fibre bundle, and the transmitted light could be detected at the end of the bundle. 

Our measurements showed that the transmitted light power of the bundle decreased in the case of compression perpendicular to the plane of the E-glass fabric, and the same was true in the case of a tensile load parallel to the fibre bundles. Because both of these loading methods cause the fibre bundles of the fabric to get closer to each other, we concluded that the loss of transmitted light power was caused by the enhanced light scattering of the fibre surfaces in contact. We proved that the change in the transmitted light power was reversible in the strain range 0%–1%, so our proposed health monitoring method can detect tensile forces if they are lower those that generate irreversible fibre breaking.

Our results indicate that an effective and inexpensive composite health monitoring technique can be developed which does not impair the integrity of the monitored structure.
